# AI-Based Drug Discovery of TKIs Targeting L858R/T790M/C797S-Mutant EGFR in Non-small Cell Lung Cancer

**DOI:** 10.3389/fphar.2021.660313

**Published:** 2021-07-28

**Authors:** Geunho Choi, Daegeun Kim, Junehwan Oh

**Affiliations:** AI LAB, AllLive Healthcare Co.,Ltd., Seongnam, Korea

**Keywords:** NSCLC, EGFR, tyrosine kinase inhibitors (TKIs), transfer learning, LSTM, virtual screening

## Abstract

Lung cancer has a high mortality rate, and non-small cell lung cancer (NSCLC) is the most common type of lung cancer. Patients have been observed to acquire resistance against various anticancer agents used for NSCLC due to L858R (or Exon del19)/T790M/C797S-EGFR mutations. Therefore, next-generation drugs are being developed to overcome this problem of acquired resistance. The goal of this study was to use artificial intelligence (AI) to discover drug candidates that can overcome acquired resistance and reduce the limitations of the current drug discovery process, such as high costs and long durations of drug design and production. To generate ligands using AI, we collected data related to tyrosine kinase inhibitors (TKIs) from accessible libraries and used LSTM (Long short term memory) based transfer learning (TL) model. Through the simplified molecular-input line-entry system (SMILES) datasets of the generated ligands, we obtained drug-like ligands via parameter-filtering, cyclic skeleton (CSK) analysis, and virtual screening utilizing deep-learning method. Based on the results of this study, we are developing prospective EGFR TKIs for NSCLC that have overcome the limitations of existing third-generation drugs.

## Introduction

Cancer is one of the leading causes of death worldwide (Sung et al., 2021). In the United States, cancer is the second leading cause of death as of 2017–2018, accounting for 21% of the deaths. The mortality rates according to the cancer type from 2014 to 2018 in the United States are as follows (rates are per 100,000 population). Lung and bronchus cancer was 38.5, breast cancer (female) was 20.1, prostate cancer was 19.0, colon and rectum cancer was 13.7, liver and intrahepatic bile duct cancer was 6.6, and Kidney and renal pelvis cancer was 3.6 (Siegel et al., 2021). Among many cancers, lung cancer has a high mortality rate. Approximately 1.7 million people died from lung cancer in 2018, with non-small cell lung cancer (NSCLC) being the cause of death in over 80% of these cases (Yuan et al., 2019). Platinum-based doublet chemotherapy (e.g., cisplatin), which directly induces cancer cell apoptosis, has been used to treat lung cancer since 2005. However, this treatment has drawbacks such as inducement of normal cell apoptosis and achievement of only a short survival period of 10 months. Since the discovery of genetic mutations related to epidermal growth factor receptor (EGFR) in 2004, numerous targeted anticancer agents with fewer side effects compared to chemotherapy have been developed. These agents have extended the survival period of patients with lung cancer to over 24 months (Li et al., 2019) (Jiao et al., 2018) (Yuan et al., 2019).

Ongoing research on targeted anticancer agents has revealed the mechanisms of various targetable pathways associated with lung cancer (e.g., EGFR, PI3K/AKT/mTOR, RAS-MAPK, and JAK/STAT). EGFR, a member of the HER family, is a transmembrane glycoprotein that regulates cell regulatory pathways involved in cell proliferation, differentiation, and apoptosis. Since the discovery of EGFR overexpression in patients with lung cancer, which revealed a correlation between EGFR tyrosine kinase expression and tumor formation, numerous agents with significant therapeutic targets in NSCLC have been developed (Le and Gerber, 2019) (Leonetti et al., 2019).

Gefitinib from AstraZeneca and Erlotinib from Roche, which are first-generation reversible inhibitors of EGFR tyrosine kinase, were approved in 2003–2013, respectively. Afatinib, a second-generation irreversible inhibitor of EGFR tyrosine kinase from Boehringer Ingelheim, was approved in 2013. These first- and second-generation drugs are used as targeted agents for NSCLC and have shown high efficacy for common activating EGFR mutations such as the L858R point mutation and exon 19 deletion. However, after a treatment period of 1–2 years, the second mutation called the “gatekeeper”, referring to the T790M-mutation in EGFR exon 20, occurs in 50–60% of patients treated with these agents, in addition to other mutations such as MET amplification and RAS mutations. The “gatekeeper” mutation reduces the effectiveness of the first- and second-generation anticancer agents by inducing drug resistance. Thus, numerous third-generation EGFR tyrosine kinase inhibitors (TKIs) sensitive to TK domain mutations (T790M) have been developed.(Jett and Carr, 2013) (Yuan et al., 2019) (Liu et al., 2018) (Grabe et al., 2018).

Osimertinib (Tagrisso) is a major third-generation EGFR TKI developed by AstraZeneca and approved by the Food and Drug Administration (FDA) in 2015. Osimertinib covalently binds (Ghosh et al., 2019) to the Cys797 residue of EGFR tyrosine kinase and is thus highly selective (Zhai et al., 2020) (Klaeger et al., 2017) and potent for the EGFR T790M mutation and other activating EGFR mutations. In 2015, however, the use of osimertinib as a third-generation EGFR TKI was shown to lead to acquired resistance, resulting from the tertiary point mutation C797S. Substitution of the Cys797 residue with serine 797 led to the loss of covalent interactions and significantly reduced drug efficacy. Consequently, fourth-generation drugs with therapeutic effects against the EGFR C797S-mutation are currently under development. (Jett and Carr, 2013) (Leonetti et al., 2019) (Grabe et al., 2018).

Drug discovery costs are increasing and research and development efficiency is decreasing (Mak and Pichika, 2019) (Scannell et al., 2012) (Schuhmacher et al., 2016). Therefore, increasing efforts have been undertaken to use artificial intelligence (AI) in drug discovery (Chen et al., 2018) (Chan et al., 2019). Unlike conventional drug discovery procedures, AI based drug discovery does not incur high experimental costs and requires only a small number of personnel.

Deep learning (Lecun et al., 2015) is artificial neural networks that mimic the brain, a complex system. Deep learning has been successfully applied to areas such as computer vision (Voulodimos et al., 2018), speech recognition (Nassif et al., 2019), and natural language processing (Young et al., 2018). Recently, studies applying AI such as deep learning to drug discovery are increasing. Researchers have developed a drug generation model using variational autoencoder (Gómez-Bombarelli et al., 2018), generative adversarial autoencoder models (Kadurin et al., 2017). A drug generation model using a recurrent neural network (RNN) architecture and reinforcement learning (Popova et al., 2018) has also been developed. Deep learning is highly sensitive to data quality and quantity. A small dataset is a bottleneck in AI-aided novel drug discovery and can be overcome by transfer learning (TL) (Segler et al., 2018) (Gupta et al., 2018) (Moret et al., 2020) (Cai et al., 2020). TL enables efficient learning even with a small amount of data.

We adopted model (Li et al., 2020) using RNN(LSTM) and TL, and conducted research with the aim of discovering 4th generation new drug candidates as L858R (or Exon del19)/T790M/C797S-mutation EGFR tyrosine kinase inhibitors related to NSCLC.

## Materials and Methods

### Data Curation and Analysis

**FIGURE 1:**
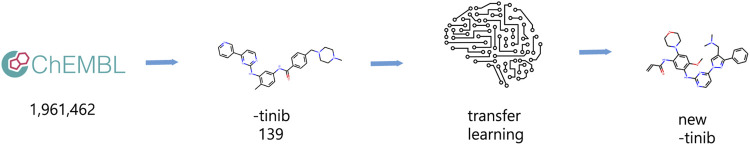
Overall process. Data preparation and generative model process.

We downloaded data for 1,961,462 compounds from ChEMBL (Gaulton et al., 2012), a curated compound database, and selected compounds whose names ended with ‘-tinib’ and additionally selected Lazertinib, creating a list of 139 compounds (Figure 1). The reason why we specifically chose ‘−tinib’ structures as our base dataset molecules from ChEMBL database is that ‘-tinib’ is an already known tyrosine kinase inhibitor that exhibits certain pharmacological effects in relation to various tyrosine kinases including our targeted protein, EGFR TK and We aim to discover promising candidates as EGFR TKIs reflecting the structural, physicochemical and biochemical features of these ‘-tinib’. The data used in the paper are available at https://github.com/cgh2797/AI_drug_discovery_EGFR. Data was input in SMILES format using the open-source cheminformatics Rdkit 2020.03.1.0. We performed a 10-fold augmentation on the ‘-tinib’ dataset as it was not large enough to train a model (Bjerrum, 2017). Additionally, we analyzed the structural similarity between compounds by examining their cyclic skeletons (CSKs) (Xu and Johnson, 2002).

### LSTM TL

Since the dataset is small and SMILES is a string format, an RNN (LSTM) TL model (Li et al., 2020) was selected. A training dataset (Li et al., 2020) was used as a base dataset, and the dataset of 139 ‘-tinib’ compounds was used as a second dataset for TL after 10-fold augmentation. The data preprocessing method was selected from the previous study (Li et al., 2020). The BasicLSTMCell function in TensorFlow was used for the two LSTM layers of the deep learning model. A dropout was applied to each LSTM layer. The keep probability was 0.8, and the number of hidden layers was 512. For the loss function, TensorFlow’s seq2seq.sequence_loss function optimized with the Adam optimizer was used. The learning rate was set to 0.003. Model training was performed using TensorFlow-gpu 1.15.0. NVIDIA GeForce RTX 2080 SUPER was used for computation.

### Filtering

Of the generated molecules, invalid molecules whose parameters could not be calculated by Rdkit were filtered out. Next, parameters including molecular weight, LogP, HBA, HBD, TPSA, and rotatable bonds were calculated using Rdkit. The weighted mean of the quantitative estimates of drug-likeness (QED) (Bickerton et al., 2012) was calculated using Rdkit. The desirability functions (d) can be described as asymmetric double sigmoidal (ADS) functions and are expressed as shown in Eq. 1. a, b, c, d, e, and f in Eq. 1 denote the parameters of the ADS function. QED is calculated by taking the geometric mean of the desirability functions multiplied by their weights *w* and can be expressed as shown in Eq. 2. Expanding the equation results in Eq. 3: This material is from our original study (Bickerton et al., 2012).

$$D\left(x\right)=a+\frac{b}{\left[1+\exp\left(-\frac{x-c+\frac{d}{2}}{e}\right)\right]}\left[1-\frac{1}{\left[1+\exp\left(-\frac{x-c-\frac{d}{2}}{f}\right)\right]}\right]$$ (1)

$${\text{QED}}_{\text{W}}=exp\left(\frac{\sum_{i=1}^{n}w_{i}\ln d_{i}}{\sum_{i=1}^{n}w_{i}}\right)$$ (2)

$${\text{QED}}_{\text{W}}=exp\left[\frac{\begin{array}{c} W_{MW}\ln d_{MW}+W_{ALOGP}\ln d_{ALOGP}+W_{HBA}\ln d_{HBA}+W_{HBD}\ln d_{HBD}\\ +W_{PSA}\ln d_{PSA}+W_{ROTB}\ln d_{ROTB}+W_{AROM}\ln d_{AROM}+W_{ALERTS}\ln d_{ALERTS} \end{array}}{W_{MW}+W_{ALOGP}+W_{HBA}+W_{HBD}+W_{PSA}+W_{ROTB}+W_{AROM}+W_{ALERTS}}\right]$$ (3)

The synthetic accessibility (SA) (Ertl and Schuffenhauer, 2009) score was calculated as a combination of two components using the Rdkit.

Sascore=fragmentScore-complexityPenalty (4)

The following screening filters were used for the parameters: 300 ≤ MW ≤ 700, 2.0 ≤ LogP≤6.0, 2.0 ≤ HBD≤6.0, 0 ≤ HBA≤12.0, HBA + HBD≤14.0, 60.0 ≤ TPSA≤140.0, and rotational bond≤12.0. All filters were applied to obtain the desirable ligands.

### AI Virtual Screening

In virtual screening, DeepDTA (Öztürk et al., 2018), a convolutional neural network-based drug target affinity prediction model, was used to predict the affinity of the candidate compounds for L858R/T790M/C797S mutant EGFR (PDB code: 6LUD). The output of the model is pK_d_ (5), which denotes the affinity between a protein and a drug.

$${\text{PK}}_{\text{d}}=-\log10\left(\frac{{\text{K}}_{\text{d}}}{1e^{9}}\right)$$ (5)

pK_d_ was predicted using Tensorflow 2.2.0, keras 2.4.3, and NVIDIA GeForce RTX 2080 SUPER.

## Results

### AI-Aided Drug Discovery

**FIGURE 2:**
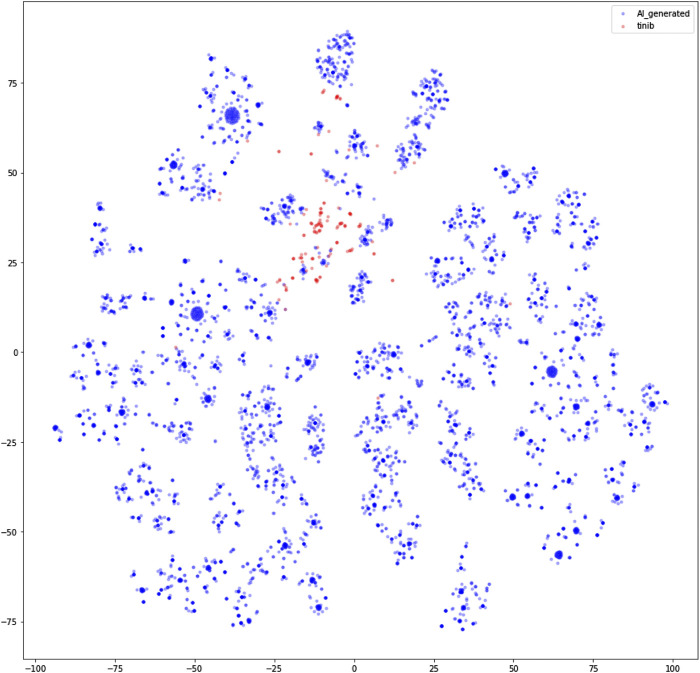
Two-dimensional representation of ‘−tinib’ and AI-generated ligands using t-SNE.

TL was used to compensate for the small quantity of ‘−tinib’ data obtained from ChEMBL. The compounds generated via TL had unique structures that were similar to the ‘−tinib’, but also exhibited the characteristics of the compounds in the training dataset. The compounds were vectorized using the Morgan Fingerprint (Cereto-Massagué et al., 2015) in Rdkit and visualized after dimensionality reduction into a two-dimensional (2D) space using t-SNE (Maaten and Hinton, 2008) in scikit-learn (Figure 2). In the 2D space, compounds with similar structures were clustered closer together, while structurally dissimilar compounds were farther from one another. The AI-generated compounds surrounded the ‘−tinib’ compounds in a ring shape. While the AI-generated compounds were similar to the ‘−tinib’ compounds based on their small distance between one another in the 2D space, they were still far enough to be considered unique, and thereby avoided patent infringement.

### Parameter Filtering

**FIGURE 3:**
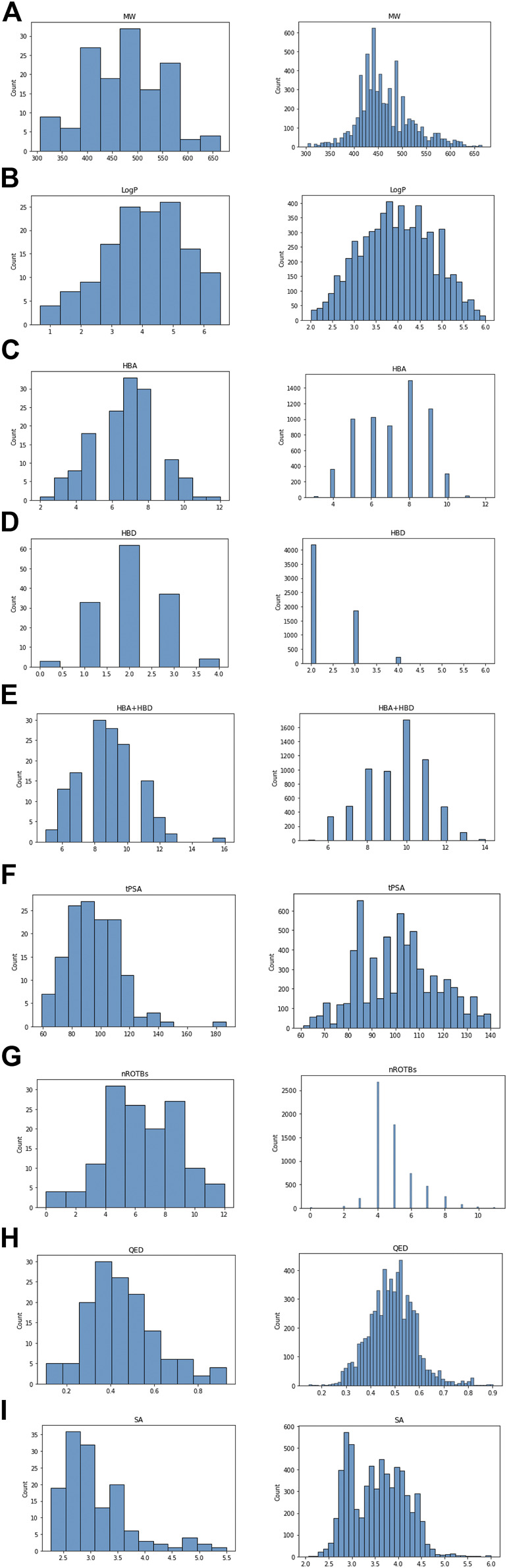
Comparison parameter distribution of ‘−tinib’ and 6,283 ligands made by AI. The figure shows the parameter distribution, the ‘−tinib’ on the left and the AI generated ligands on the right. **(A)** Molecular Weight, **(B)** LogP, **(C)** HBA, **(D)** HBD, **(E)** HBA + HBD, **(F)** tPSA, **(G)** Rotatable bond, **(H)** QED, and **(I)** SA (Synthetic Accessibility) score.

Approximately 20% of 10,316 AI-generated ligand SMILES were invalid SMILES that did not meet the encoding rules and were thus removed. Following the removal, the remaining ligands were screened by filtering based on MW, LogP, TPSA, HBA, HBD, HBA + HBD, and rotatable bonds to ultimately remove undruggable molecules. As a result of screening using parameter filtering, we obtain 6,283 ligands out of 10,316. After that, the distributions of parameters such as MW, LogP, TPSA, HBA, HBD, HBA + HBD, rotatable bond, QED, and SA were compared between the AI-generated and ‘−tinib’ compounds (Figure 3). The parameters showed highly similar distributions between the two groups of compounds. The physicochemical characteristics of the existing drugs were well-reproduced by the AI-generated compounds.

### Structural Similarity Based on CSK

**FIGURE 4:**
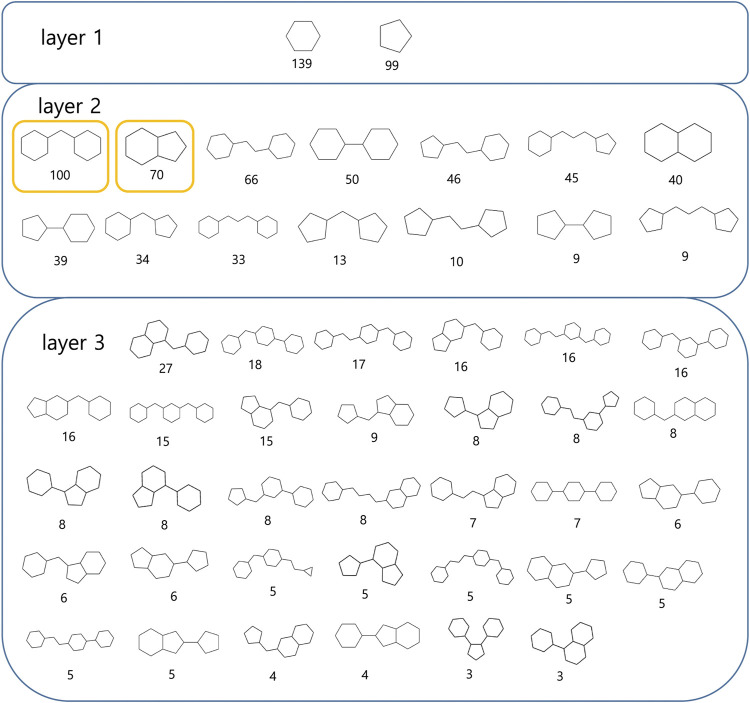
Most frequent CSK from ‘-tinib’.

A scaffold is the core structure of a compound. CSK is an abstract version of a scaffold. We examined pharmaceutically meaningful structural similarities between ‘-tinib’ by computing CSKs to select drug-like compounds. We created a hierarchical figure by placing structures with a single ring in layer 1, those with two rings in layer 2, and those with three rings in layer 3 (Figure 4).

Bridged-bicyclic rings and fused-bicyclic rings were the two most commonly observed types of CSK, each, with a count of 100 and 70 (marked with yellow). To assess the reproducibility of these results, we analyzed the CSKs of 6,283 molecules generated from the training dataset of 139 ‘−tinib’ compounds using a machine learning model. The 3,308 and 2,254 ligands had bridged-bicyclic rings and fused-bicyclic rings, respectively, and thus, the results were deemed reproducible. We confirmed the structural similarities between the original and AI-generated ligand groups based on CSKs.

### AI Virtual Screening

**FIGURE 5:**
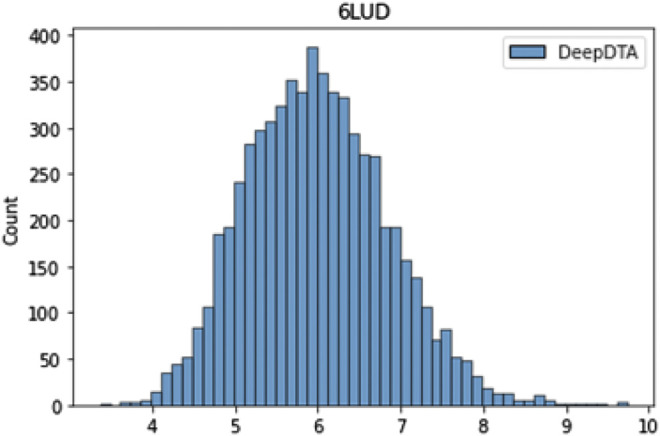
Drug target affinity score distribution predicted by DeepDTA.

We used DeepDTA, a machine learning-based model, for fast virtual screening of druggable ligands based on their target binding affinity for L858R/T790M/C797S mutant EGFR (PDB code: 6LUD). Figure 5 shows the affinity distribution predicted by DeepDTA. Ligands with high ${\text{pK}}_{\text{d}}$ score were predicted to have high affinity for the L858R/T790M/C797S mutant EGFR.

### Screening Ligands Based on Stringent Criterion

To extract ligands for docking simulation and non-clinical experiments, we screened ligands using stringent criterion. As a result, 360 ligands that we can calculate by docking program were selected. More information on 360 ligands is available at https://github.com/cgh2797/AI_drug_discovery_EGFR.

## Discussion

In this study, we used AI-based drug discovery to overcome the issues of high cost and low efficiency of drug research and development at present. We used AI to discover drug-like ligands resembling TKIs associated with EGFR in NSCLC and screened the candidates through the following process.

We extracted 139 ligands associated with TKIs from approximately 1.96 million compounds in ChEMBL. Next, we performed deep learning using an RNN (LSTM) to generate 10,316 SMILES associated with TKI molecules. Through parameter-filtering using in-house methods, we narrowed down the SMILES to 6,283 drug-like ligands with affinity for L858R/T790M/C797S mutant EGFR in NSCLC. To gain additional understanding of the selected ligands, we analyzed their CSKs to examine the structural similarity between the AI-generated molecules and the existing ‘−tinib’ from ChEMBL. We used a deep learning model such as DeepDTA to predict the binding affinities of these compounds for L858R/T790M/C797S mutant EGFR. Finally, by applying stringent criterion, 360 ligands were obtained.

However, there are several limitations to this study. First, it is difficult to create only covalent ligands or determine if the ligands are covalent, when generating various ligands through AI methods. Since we based our results on Osimertinib, which is a representative covalent TKI, obtaining covalently binding ligands is also an ideal aim in this study. Thus, in a follow-up study, we will select promising compounds by determining directly based on scientific rationale whether the 360 ligands are “covalent” or “noncovalent” ligands using an in silico docking prediction method. Second, there are selectivity issues, which should be addressed even within the tyrosine kinase family. Since our research is in its early stage, our primary goal of the research is to preferentially discovery candidates as EGFR TKIs with notable efficacy (i.e., binding affinity). After identifying promising candidates, a more detailed research would be conducted to resolve the selectivity issues. Finally, another limitation of this study is that although new druggable ligands were found, experiments such as in silico docking, synthesis in the laboratory, and preclinical trials were not conducted. Hence, further studies on improving the therapeutic potential of our selected ligands, such as in silico docking prediction, synthesis in the laboratory, and preclinical trials (e.g., efficacy and safety trials), would be undertaken. Accordingly, a more detailed research would also be conducted to resolve the aforementioned tyrosine kinase selectivity issues (e.g., SAR by structural modification).

We don’t put meaning to simply discover new druggable ligands similar to existing ‘−tinib’ using AI. Since AI has infinite potential for applications in drug discovery, our goal is not only limited to drug discovery, but also includes the successful development of drugs that can receive FDA approval.

Therefore, our next task is to discover new candidates with good drug-like profiles (efficacy, toxicity, pharmacodynamics, pharmacokinetics, etc.) and identify those eligible for drug approval. We must also explore the possibility of using these compounds in combination therapy. By presenting examples of successful new drug development through these series of processes, new drug development technology using AI will become a new drug discovery paradigm.

## Data Availability

The original contributions presented in the study are included in the article/Supplementary Material, further inquiries can be directed to the corresponding author.
